# Conversations About Climate Risk, Adaptation and Resilience in Africa

**DOI:** 10.1007/978-3-030-61160-6_9

**Published:** 2021-01-20

**Authors:** Declan Conway, Katharine Vincent

**Affiliations:** 1 Grantham Research Institute on Climate Change and the Environment, London School of Economics and Political Science, London, UK; 2 Kulima Integrated Development Solutions, Pietermaritzburg, South Africa; 3 Grantham Research Institute on Climate Change and the Environment, London School of Economics and Political Science, London, UK; 4 Kulima Integrated Development Solutions, Pietermaritzburg, South Africa

**Keywords:** Climate risk, Adaptation, Climate information, Co-production

## Abstract

This book contributes to previous and ongoing action to initiate and inform conversations about climate risk and the need for adaptation and resilience building. This involves blending insights from climate science about what the future climate will look like with experiences of the social science of response through adaptation, based on practical applications in a variety of contexts. In this chapter, we reflect on these conversations and what they mean for the growing adaptation agenda. We consider who needs to be involved in conversations about adaptation, how such conversations can be structured and the need to assess their outcomes. We profile important considerations relevant for tailoring climate information to make adaptation decisions and discuss the outcomes of different types of conversations. We conclude by noting the significance of recent major climate events and the rapidly evolving risk landscape in sub-Saharan Africa, and arguing that the need for these conversations is ever more evident. The experiences outlined in this book provide a starting point for conversations about adaptation that aim to inform future action.

## Introduction

Climate change is posing a risk with which we all have to live. As a result, the learning process about climate change adaptation will continue for decades into the future. There are no blueprints for this process: instead we have to learn by trial and improvement.

In Chap. 10.1007/978-3-030-61160-6_1, we introduced a series of questions that informed the writing of the subsequent chapters, based on our collective insights about what are important considerations for adaptation and resilience:

- What are the characteristics of the decision problem and how are they defined and by whom?
- What kinds of interactions occur and who is involved?
- What are the key contextual factors including the significance of historical climate risks and role of institutions and governance?
- How are climate risks characterised and communicated, over which timescales?
- To what extent does uncertainty about climate feature in the analysis?
- To what extent are non-climate considerations important and how they are addressed?
- What are the reflections—what works well and why?

Reflecting on the resulting chapters, which address the questions in a variety of ways (and sometimes implicitly), we see their collective contribution as adding to previous and ongoing action to initiate and inform conversations about climate risk and the need for adaptation and resilience building. This involves blending knowledge from climate science that provides insights into what the future climate will look like with experiences of the social science of response through adaptation, based on practical applications in a variety of contexts in sub-Saharan Africa. To do so requires a process of communication. By raising awareness, sharing knowledge between different actors and promoting inclusion, the book aims to ‘inform the conversation’ that is ongoing in international and national policy arenas, and more broadly in society, to help make more equitable and effective decisions to reduce climate risk. We therefore structure this chapter around the idea of conversations that occur in support of adaptation and resilience in the face of climate risk.

Much of this book deals with underlying principles and different structures designed to facilitate effective conversations along the whole climate services value chain, which includes the robustness of information, approaches to engagement and construction of knowledge. Climate change is defined as a wicked problem—namely one that defies easy resolution due to constantly changing baselines and inherent uncertainty. This means that addressing it requires post-normal science, where science cannot be divorced from the values and norms that give it value and use (Funtowicz and Ravetz 1993). To take those values and norms into account, post-normal science requires active engagement of the non-scientific communities. Participatory engagement and co-production are among the mechanisms through which post-normal science takes place. Equity and inclusion are core principles to be promoted through these approaches, taking into account who is involved and their roles. These structured conversations are also designed to address in various ways factors that are required for succesful information use, including credibility, legitimacy/ trust and salience, among others (Cash et al. 2003; and running through Chaps. 10.1007/978-3-030-61160-6_1–10.1007/978-3-030-61160-6_8).

This chapter reflects on these conversations and what they mean for the growing adaptation agenda. In the next section, we consider who needs to be involved in conversations about adaptation before turning to ways in which such conversations can be structured and the need to assess their outcomes. We then examine what considerations are relevant for tailoring climate information to make adaptation decisions. In the following section, we reflect on the outcomes of those conversations, and we then conclude with a section about focusing conversations on the need for action.

## Who Is or Needs to Be in the Conversation?

**Fig. 9.1:**
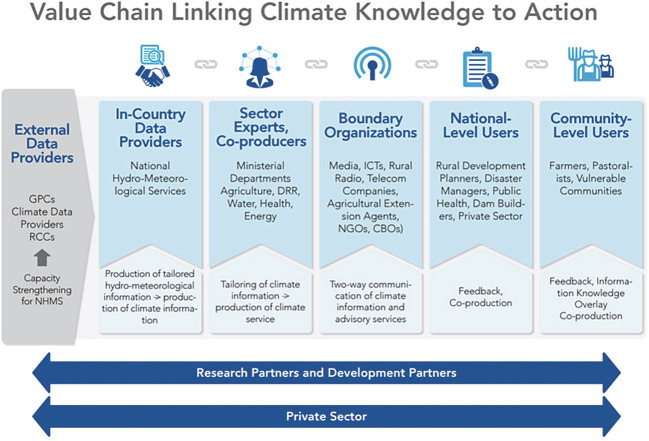
The range of actors and roles involved along the climate services value chain linking knowledge to action. Source: Figure prepared by Winrock International and WMO for the USAID-supported Assessing Sustainability and Effectiveness of Climate Information Services in Africa project. Washington, DC, USA; https://gfcs.wmo.int//saly-coordination-workshop

Conversations about adaptation need to engage widely. Figure 9.1 from the Global Framework for Climate Services (GFCS) shows the range of actors and roles involved along the climate information value chain linking knowledge to action. The climate science community is a crucial part of the process—this is where new understanding is generated that has great potential for application. In much of Africa, National Meteorological and Hydrological Services (NMHS) and regional economic communities and their associated bodies (the African Center of Meteorological Application for Development—ACMAD, the Intergovernmental Authority on Development Climate Prediction and Application Center—ICPAC, and the Southern African Development Community Climate Service Centre—SADC CSC) hold formal mandates for collecting data, issuing forecasts and reporting on climate. This occurs through the mechanisms of, for example, the pan-African inter-governmental bodies such as the African Ministerial Conference on Meteorology (AMCOMET), the African Ministers’ Council on Water (AMCOW), the African Ministerial Conference on the Environment (AMCEN) and Regional Climate Fora, as well as internationally through the United Nations Framework Convention on Climate Change (UNFCCC ) and the World Meteorological Organisation (WMO ). They are where most of the technical capacity exists, complemented by the university sector where, with some exceptions, capacity is generally more limited than the global North. Many institutions support only a few or just one individual with some experience, although the number is growing. In Future Climate for Africa (FCFA ), the consortia integrated some of these organisations either as core partners or through regular engagement.

As this book clearly shows, the conversations necessary for adaptation go way beyond the science. This can create new and far-reaching demands on the traditional science-based organisations and their staff—in terms of remit and skills and financial resources. The gap between core science and application can be vast, and it does not need to engage everyone, indeed for many scientists this is unnecessary. A growing range of boundary organisations are also concerned with adaptation and resilience and have expanding numbers of specialists, and thus play a critical role within the climate services value chain. In some cases, very limited technical information is actually required to achieve confidence in making decisions. In fact, leaving climate information completely out of the conversation initially may be good practice to avoid priming and to openly identify primary concerns (e.g. Chap. 10.1007/978-3-030-61160-6_2).

Important questions arise with respect to whether and how the established leading science organisations take on these new roles and to what extent the necessary knowledge and skills are presently being taught. And are they even the right people or organisations to take on these roles? While it is beyond our scope to answer definitively, experience suggests that it is difficult for NMHS to engage without additional funding, broader staff capacity, or changes to their mandate. There is a grey area between official responsibilities as data providers and demands to work with sector experts and users, and roles for intermediaries who require access to data and people’s time (providers, sector experts and users). The interfaces or boundary areas between the data providers and sector producers (Fig. 9.1) need more recognition and formal guidance on roles and responsibilities, backed up by allocation of resources and capacity strengthening to deliver results.

University teaching offers the most promise to fill the skills gap over the long-term and could benefit from modifying existing courses/modules and setting-up new interdisciplinary degree programmes. There are well-known drawbacks to short-term training programmes and technical assistance (Mataya et al. 2019). Placements and collaborations through linking student dissertation topics with practitioner organisations offer a route for trans-disciplinary collaboration between researchers and practitioners and the potential for deeper understanding of decision contexts (Chap. 10.1007/978-3-030-61160-6_6). The Future Resilience for African Cities and Lands (FRACTAL ) programme found very positive experiences through embedded researchers working in host organisations helping to broker multi-stakeholder engagements and sustain momentum (Chap. 10.1007/978-3-030-61160-6_7). These efforts are designed to address concerns about externally and technically driven agendas. They aim to support initiatives from the ground up—promoting more endogenously African driven actions (Vogel et al. 2019). This includes working hard to raise the low presence of African-based researchers in academic journals and Intergovernmental Panel on Climate Change (IPCC ) reports (Pasgaard et al. 2015) and giving more credence and platforms to alternative ways of sharing insights, recognising different forms of knowledge. Building confidence through long-term commitment will help (Hewitson 2015). Similarly, and crucially, so will strengthening the opportunities for early career researchers as the next generation who can bring their insight and voices to the challenge (Mustelin et al. 2013).

Taking the time to understand contexts at any scale of decision-making highlights that it is never just about the climate. Climate impacts play out against pre-existing exposures and vulnerabilities, reactive or anticipatory responses are conditioned by people’s capacity and longer-term planning, both of which reflect the underlying power structures that operate through governance within societies. Such structures play a major role in determining processes in decision-making and reflect and reproduce the underlying socio-political patterns that construct vulnerability, a context that extends far beyond adaptation as narrowly defined by a focus on climate risk (Adger et al. 2009). Careful design and deep engagement that recognises these contexts should underpin approaches to adaptation. Involving national level and community level users is fundamental to this process.

## How Have These Conversations Taken Place?

The cases presented in earlier chapters exemplify various ways in which conversations can be inclusive. Co-production is a process that brings together different parties and, as Vincent et al. (Chap. 10.1007/978-3-030-61160-6_3) note, is increasingly promoted to enhance the utility and usability of climate information. They proposed ten principles of co-production derived from both academic literature and practical experiences but, crucially, emphasised that there is no blueprint for what is required. Participatory Impact Pathways Analysis (PIPA) comprises problem tree analysis, visioning and stakeholder mapping, and a climate information training workshop, leading to an options matrix that aims to support preparedness (Chaps. 10.1007/978-3-030-61160-6_3 and 10.1007/978-3-030-61160-6_4). Participatory Scenario Planning (PSP) takes place through consultative dialogue between weather and climate information producers and users who generate sector-specific advisories (Chap. 10.1007/978-3-030-61160-6_5). Chapter 10.1007/978-3-030-61160-6_7 outlines how multiple fora in the FRACTAL programme served to enhance interaction with stakeholders in urban settings and ensure that the problem context drives the construction of climate information, including learning labs and embedded researchers. These fora helped inform and refine the interactive and inclusive process of information distillation and Climate Risk Narratives outlined in Chap. 10.1007/978-3-030-61160-6_2.

Conversations have diverse and often intangible outcomes within and through the climate services value chain. Growing investment in adaptation and a scaling up of the types of approaches presented here underscores the need to define and collect evidence of effective outcomes. Dialogue and co-production are time consuming, they often require physical meetings and involve many people, all of which generate significant costs and are increasingly hampered by issues of fatigue in some frequently targeted user groups. NMHS and national and community level organisations are likely to require extra resources for the new demands on staff time that will arise when tasked with adaptation, many of whom may also need knowledge and skill sharing. However, applications of co-production on climate change timescales in Africa are in their infancy and there is a need for more extensive assessment of their utility and the potential for replication and scaling up (Wall et al. 2017). As well as challenges with evaluating co-production, universal indicators for adaptation are unrealistic given context-dependent risk (Leiter and Pringle 2018) and differences in people’s values as they relate to their experience and sense of place that are largely intangible and non-commensurable (Tschakert et al. 2019). Nevertheless, we see a need for more comparative analysis with a focus on locally or self-defined measures of outcomes, cost effectiveness, strengths and weaknesses of approaches, processes and outcomes, and potential for replicability at scale. The rich history of experience in other fields of application in co-production and participatory approaches in development could offer some useful lessons. Evidence is now emerging from Community-Based Adaptation (CBA) projects. For example, an assessment of whether CBA was effectively promoting adaptive capacity in 32 projects across four Pacific Island countries found mixed performance, with positive responses for appropriateness but issues highlighted for sustainability (McNamara et al. 2020).

## What Are Conversations Based On?

A major part of FCFA focused on high-level climate science, producing many articles on Africa’s climate. These include uncovering positive trends in daily rainfall intensity in West Africa (Taylor et al. 2017), proposing a framework for an African lens in climate model analysis (James et al. 2018), and improvements in simulation of crucial convective-scale processes through high resolution modelling (Kendon et al. 2019) together with the first climate projections at this scale. While this vast body of work builds the science knowledge base that interfaces with applications, the pathways to impact can take many years. The types of climate information used in this book are diverse, representing multiple timescales and in all cases a substantial tailoring of information that addresses to varying degrees its credibility, legitimacy and salience. Chapters 10.1007/978-3-030-61160-6_4 and 10.1007/978-3-030-61160-6_5 consider seasonal forecasting (Audia et al. and Tembo-Nhlema et al., this volume), two adopt a Climate Risk Narrative approach covering medium term future timescales informed by climate model projections (Chaps. 10.1007/978-3-030-61160-6_2 and 10.1007/978-3-030-61160-6_7), and two use elements of an impact-led approach by simulating impacts directly with climate model projections and process models (Chaps. 10.1007/978-3-030-61160-6_6 and 10.1007/978-3-030-61160-6_8). Development of Climate Risk Narratives draws heavily on the science but may result in summaries or infographics (Burgin et al. 2019) that bypass the technical aspects, underscoring a case for involving stakeholders in this translation process (Chap. 10.1007/978-3-030-61160-6_2).

Of note is that there is little detail about the climate information itself—the focus is primarily on the process of reaching a point where information becomes usable. Apart from Chap. 10.1007/978-3-030-61160-6_2, which actively promotes making the value judgements in the climate science explicit in a consultative process, and Chap. 10.1007/978-3-030-61160-6_6, which presents a technically detailed approach to decide on which model projections to use, climate models are barely mentioned. By intent, there is limited description of greenhouse gas emissions scenarios, whether and how to downscale from coarse to fine climate model resolution information, quantification of uncertainty about changes in climate variables, and discussion of how many and which climate models to use. These are all stages in the cascade of uncertainty from global climate projections to local or decision-relevant scales of information that often form core content in climate impact studies (e.g. Wilby and Dessai 2010; Wilby et al. 2009). Moreover, in most of the chapters, there is a strong focus on understanding recent and current experiences of climate risk to make the issue more tangible and relevant to decision-makers. While the chapters address the interface between top-down and bottom-up approaches, they tend to draw more heavily from elements of the latter, avoiding the technical complexity because it may not be of direct value to actual decisions underway (Conway et al. 2019). In short, there are various ways of facilitating conversations that can take place with currently available information to manage climate risk and facilitate adaptation and resilience—as outlined in Chaps. 10.1007/978-3-030-61160-6_3–10.1007/978-3-030-61160-6_8.

## Considerations Relevant for Tailoring Climate Information to Make Adaptation Decisions

Deciding on how to frame the elements of adaptation, particularly the climate information requirements, requires consultation to establish the aim of the exercise, for whom, and what are their main concerns? A key factor in this is the scale of the decision. While adaptation has been widely seen as a local and place-specific process there are many situations where decisions have large areal dimensions with consequences far into the future. There is also growing recognition that climate risks cross boundaries (jurisdictional, political and sectoral) and as such adaptation action can redistribute or transfer climate risks (Benzie and Persson 2019). This raises questions about the scale of the adaptation response space—we aim to be comprehensive, but how to do so without making the decision context too complex and paralysing the process, with too many options and actors?

Many forms of climate information and ways of structuring conversations about climate risk and adaptation are available to suit the scale of the decision situation. Most involve variations of a sequence of actions that include: consulting about the problem and agreeing the aims of the exercise; developing an understanding of the system of interest; identifying what is important for stakeholders, assessing the significance of future climate risks to development plans and identifying options (e.g. Willows et al. 2003). Further stages can include implementing decisions, followed by monitoring, evaluating and adjusting. We stress that simplicity is essential to ensure sustained use. While more sophisticated and ambitious use of climate information, tools and decision support systems are attractive and promoted by researchers, they often fail to meet the realities of operational practice. For example, Clar and Steurer (2018) identified 88 support tools for climate services/adaptation but they had received very limited evaluation. When they examined whether and how the Willows et al. (2003) framework, a tool that had been widely promoted in the UK during the late 1990s and 2000s, was used by local authorities, they found very low levels of awareness and use.

**Fig. 9.2:**
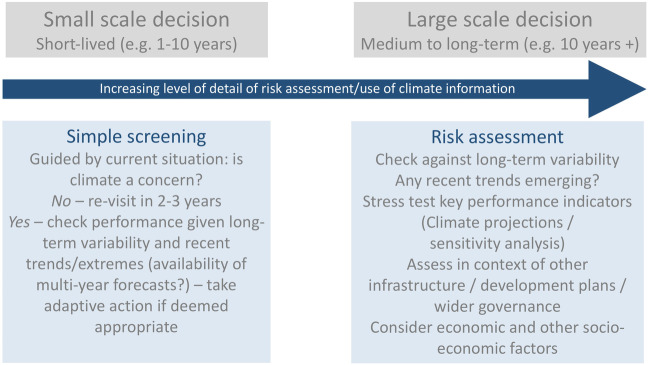
The types of climate information and approach vary with the scale of the decision situation

Deciding which approach is most suitable is an important initial part of an adaptation process and requires consultation. Bearing this in mind, Fig. 9.2 shows a continuum ranging from a simple light touch approach suitable for many small and short-lived decisions through to detailed assessments for major long-lived decisions such as large-scale infrastructure and urban planning. For light touch screening approaches such as adjustments to agricultural practices and technology, or selecting small-scale water and sanitation technologies, only limited climate information is required. A summary of recent variability can be extrapolated into the next few years, flagging the need for action where any crucial climate sensitivities have been experienced, and if not, reviewing the situation every two to three years.

However, major decisions require careful planning and risk assessment should include climate. Where large investments are involved and their planned lifespan is long and decisions may be irreversible, it is crucial to consider future climate risk. While examples are emerging of climate risk assessments, they are still far from routine. Chapter 10.1007/978-3-030-61160-6_6 describes part of a detailed climate risk analysis for a major dam project in the Rufiji River Basin in Tanzania that would amount to a considerable budget (many thousands of US dollars), but this is small compared to the cost of the infrastructure and the potential costs of future underperformance. It is on longer-term future timescales that uncertainty about the future climate and other socio-economic factors have more bearing on the risk assessment process. Deriving climate information can easily become bogged down in technical detail, be capacity and resource intensive and lead to confusing messages about uncertainty. Detailed assessments should not be undertaken lightly.

What we can say with high confidence is that warming trends will continue, increasing the frequency and intensity of heatwaves and, other things being equal, enhancing soil moisture deficits. For rainfall, ideas of Robust Decision-Making (RDM) or Decision-Making Under Uncertainty (DMUU ), like the principles captured in the Flexible, Robust, Economic no/low Regrets and Equitable framework (FREE, Chap. 10.1007/978-3-030-61160-6_4), can help address the uncertainty . Such approaches are designed to identify decisions and adaptation options that work reasonably well across large ranges of uncertain future climatic conditions or that retain flexibility in a cost-effective manner (Groves and Lempert 2007). These principles can be applied at a range of decision scales with limited inputs.

## What Have Been the Outcomes of These Conversations?

FCFA was designed out of a realisation that many sub-Saharan African countries do not include climate information in medium- to long-term planning. The programme aims were framed as: achieving better tailoring of information to needs; greater recognition of political factors in decision-making; and more consideration of ethical dimensions of promoting long-term climate risk in situations dominated by pressing developmental priorities and short-term political timeframes (Jones et al. 2015). Despite the commencement of new conversations and application of new tools and decision-making frameworks to increase use of climate information, there remain, to our knowledge, limited instances where direct use of information on medium to long-term future climate change routinely form part of formal decision processes in sub-Saharan Africa.

Most of our examples are discrete, where specific research projects have engaged with formal and informal agencies and their planning processes, which have their own lock in effects and path dependency. In some cases, the influence may be evident. For example, in Chap. 10.1007/978-3-030-61160-6_7 the authors were able to work closely with policymakers in preparation of the City Council’s Strategic Plan (2017–2021) and through multi-level engagement in Uganda; Chap. 10.1007/978-3-030-61160-6_8 fed insights into district level budget decisions and revisions to the National Environment Bill. In many other cases, the influence is less distinct but still present through the sharing of information between those involved in the engagements (many more specific examples can be found at FCFA, https://futureclimateafrica.org/).

## Focusing Conversations on the Need for Action

FCFA is one of many programmes that are contributing to an ongoing conversation about climate change, adaptation and resilience building—gradually making society more climate aware, more climate literate, and more climate prepared. And the need for those conversations has become ever more evident in the four years since the start of the FCFA programme. During that time Africa has experienced, alongside many other extremes, one of the strongest El Niño–Southern Oscillation events in over 50 years (in 2015–16) and the strongest Indian Ocean Dipole event in six decades (2019). Both are large-scale modes of global climate variability that influenced conditions over extensive areas of Africa and brought with them wide-ranging impacts. The drought in southern Africa that accompanied the 2015–16 El Niño–Southern Oscillation resulted in an extensive loss of crops and livestock and an increase in food prices, driving an estimated 39 million people into deeper food insecurity (Archer et al. 2017). The Indian Ocean Dipole was associated with high rainfall across large parts of East and the Horn of Africa between October and November in 2019. Heavy rainfall caused landslides and flash floods with millions of people affected. The Day Zero water supply crisis in Cape Town received global media coverage, prompting intense debate over the role of drought and water resource management decisions and infrastructure prior to and during the crisis (e.g. Taing et al. 2019). The event exemplifies how poverty mediates the ways in which exposure translates into impacts, the complex and often contested causal pathways between climate hazards and their human consequences, and the windows of opportunity for learning and policy response that extreme events provide.

Future risk is likely to lie well beyond what has been experienced in the recent past and, however well intentioned, levels of concern and funding for contingency plans fade over time. We are heading for minimum 1.5 °C and quite possibly well above 2 °C of global mean temperature rise this century. The impacts of changing frequency and intensity of extremes along the way could be exacerbated by the potential for exceeding low probability but high-impact tipping points (Lenton et al. 2008). New landscapes of risk are emerging as a result of hazard complexes occurring in rapid succession or in cascades through knock-on effects across sectors. For example, the 2015–16 drought in southern Africa was associated with complex compound features: successive years with low rainfall and extreme temperatures leading to rain-fed crop failure and heat stress on livestock and plants; reduced river flows leading to less irrigation and less hydropower; cascading to further impacts on water pumping for urban and irrigation uses and health/ hygiene problems (Chap. 10.1007/978-3-030-61160-6_7; Gannon et al. 2018). At the time of writing, the impacts of Covid-19 are playing out against the compound effects of flooding in East Africa in early 2019 and massive locust infestations, both associated with the extreme Indian Ocean Dipole of 2019 (Marsham 2020).

Fortunately, the increasing evidence of climate risk is accompanied by two important and related drivers of growing demand for adaptation measures. The first is greater recognition and experience of the escalating social and economic burden caused by changing frequencies, intensities and combinations of hazards. The second is stronger international and national level policy commitments, such as the Paris Agreement and the Global Goal on Adaptation. There is competition for resources and development priorities and short-term political and planning horizons, but by emphasising the need to embed the climate dimension in a wider context of decision-making we recognise the importance of aligning adaptation and resilience building with other sectors and actors, to make the agenda relevant and tractable for policy and practice. In short, conversations about climate need to be had. Our premise is that the interface between experiential and policy drivers of autonomous and planned adaptation and resilience building stimulates innovation for practice. It is at this interface that the value and use of climate information is raised and tested. And the experiences outlined in this book provide a starting point for such conversations to inform future action.
